# Iron, anemia and hepcidin in malaria

**DOI:** 10.3389/fphar.2014.00125

**Published:** 2014-05-30

**Authors:** Natasha Spottiswoode, Patrick E. Duffy, Hal Drakesmith

**Affiliations:** ^1^Laboratory of Malaria Immunology and Vaccinology, National Institute of Allergy and Infectious Diseases, National Institutes of HealthBethesda, MD, USA; ^2^MRC Human Immunology Unit, Weatherall Institute of Molecular Medicine, University of OxfordOxford, UK

**Keywords:** hepcidin, malaria, iron, anemia, global health

## Abstract

Malaria and iron have a complex but important relationship. *Plasmodium* proliferation requires iron, both during the clinically silent liver stage of growth and in the disease-associated phase of erythrocyte infection. Precisely how the protozoan acquires its iron from its mammalian host remains unclear, but iron chelators can inhibit pathogen growth *in vitro* and in animal models. In humans, iron deficiency appears to protect against severe malaria, while iron supplementation increases risks of infection and disease. Malaria itself causes profound disturbances in physiological iron distribution and utilization, through mechanisms that include hemolysis, release of heme, dyserythropoiesis, anemia, deposition of iron in macrophages, and inhibition of dietary iron absorption. These effects have significant consequences. Malarial anemia is a major global health problem, especially in children, that remains incompletely understood and is not straightforward to treat. Furthermore, the changes in iron metabolism during a malaria infection may modulate susceptibility to co-infections. The release of heme and accumulation of iron in granulocytes may explain increased vulnerability to non-typhoidal Salmonella during malaria. The redistribution of iron away from hepatocytes and into macrophages may confer host resistance to superinfection, whereby blood-stage parasitemia prevents the development of a second liver-stage *Plasmodium* infection in the same organism. Key to understanding the pathophysiology of iron metabolism in malaria is the activity of the iron regulatory hormone hepcidin. Hepcidin is upregulated during blood-stage parasitemia and likely mediates much of the iron redistribution that accompanies disease. Understanding the regulation and role of hepcidin may offer new opportunities to combat malaria and formulate better approaches to treat anemia in the developing world.

## INTRODUCTION

Among the many nutrients required for human survival, iron plays a unique role in determining disease susceptibility. This relationship is especially well studied in the case of malaria. Parasites of the genus *Plasmodium* cause malaria, although “malaria” is generally used to refer to symptomatic *Plasmodium falciparum* infection, unless otherwise specified. Malaria is currently one of the most geographically widespread and deadly diseases, and responsible for the deaths of an estimated 600,000 people per year (Malaria World Report, 2013^1^). The controversial relationships between malaria and iron have been the subject of widespread discussion and debate by the global health community since 2006, when a randomized large-scale trial on the island of Pemba found that iron supplementation in children was linked with an increase in malaria-related mortality (Sazawal et al., 2006). Two Cochrane reviews (Ojukwu et al., 2009; Okebe et al., 2011) published subsequent to this finding have not resolved this conundrum for physicians and policymakers. Furthermore, while iron supplementation appears to be linked with increased malarial mortality, malaria infections are a major global cause of anemia (Kassebaum et al., 2014); and measures taken to decrease malaria at a population level also decrease anemia (Meremikwu et al., 2012).

The interactions between malaria and iron have only lately begun to be understood at the molecular level. Primarily, the discovery of the iron regulatory hormone hepcidin has given us new understanding of human iron physiology and pathophysiology. Hepcidin serves to block iron absorption from the diet and also to route iron in the body into macrophages and away from the serum. Hepcidin plays a complex but vital role in both the iron restriction that occurs during malaria infection, and in determining iron status and thereby influencing disease susceptibility. In this review, we examine the known interactions between physiological iron deficiency or repletion, iron supplementation, malaria, and hepcidin, and offer recommendations and suggestions for future work.

## IRON DEFICIENCY PROTECTS FROM MALARIA INFECTION

Variations in the iron levels of susceptible hosts may modulate the frequency and clinical severity of malaria infections. Gwamaka et al. (2012) collected detailed data from a large cohort of Tanzanian children (birth – 3 years). In this vulnerable population, iron deficiency at healthy aparasitemic visits was strongly associated with decreased future risk of parasitemia and severe malaria (Gwamaka et al., 2012). Iron deficiency in this study was defined by low ferritin (<30 ng/mL) in individuals with low C-reactive protein (CRP). A higher ferritin cutoff (<70 ng/mL) was used to define individuals with higher CRP as iron deficient; plasma ferritin is considered to be representative of iron stores in healthy individuals but increases acutely in infections.

A further study in a slightly older cohort of Kenyan children (8 months–8 years) found that iron repletion (defined as ferritin ≥ 12 ng/mL, with transferrin saturation ≥10%, children with high CRP excluded), was predictive of clinical malaria episodes in the year following measurement (Nyakeriga et al., 2004). Separately, Jonker et al. (2012b) noted a similar effect in Malawian children (6 months–5 years): iron-deficient children (defined as plasma ferritin <30 ng/mL), had a lower incidence of clinical malaria the subsequent year.

Studies performed in pregnant women also may indicate an association between iron deficiency and protection from clinical malaria, although somewhat less data are available. Two cross-sectional studies have found that at the time of delivery, placental malaria was associated with iron replete status (Kabyemela et al., 2008; Senga et al., 2011). A limitation of this approach is that all currently used measures of iron status, such as ferritin, can be distorted by inflammation and infection, and thus cross-sectional studies may be of limited utility in understanding this relationship. Only one study has so far attempted to examine the predictive values of iron status on future placental or peripheral parasitemia in pregnant women (Senga et al., 2012). This study measured iron status by examining zinc protoporyphyrin (ZPP) levels. At both the first antenatal visit and at delivery, levels of ZPP indicative of iron repletion were associated with parasitemia; however, analyzing these data for ZPP as a predictive measure were complicated by the elevation of ZPP by concurrent parasitemia. The authors concluded that in this population, with a high incidence of parasitemia and associated inflammation, ZPP alone is not a valid measure of iron status, a concern that also could be applied to pediatric populations. Although the available data suggest a relationship, further studies that recruit women early in pregnancy, stratify carefully by gravidity and malaria transmission intensity, and follow their outcomes closely are required to definitively answer the question of whether iron status in women can predict malaria risk, as in pediatric populations.

## IRON SUPPLEMENTATION AND MALARIA INFECTION

While iron deficiency appears to offer some protection against malaria, iron supplementation may increase the vulnerability of susceptible populations to infection. Consequently, the use of population-scale iron supplementation in malaria-endemic areas is currently highly controversial. Early studies postulated a link between oral intake of iron and malaria susceptibility (Murray et al., 1978), as exemplified by flare-ups of latent infections following refeeding (Murray et al., 1975; Murray and Murray, 1977) or differing malaria susceptibility associated with different diets (Murray et al., 1980). In 2006, the “Pemba trial,” a large-scale, randomized trial of iron supplementation in an area with very high malaria transmission, was stopped prematurely when trial monitoring boards found a link between the supplementation of children with iron and folic acid and subsequent malaria infection and mortality (Sazawal et al., 2006). Several studies that followed the Pemba trial produced results that appeared to contradict this finding, showing either no association between supplementation and malaria risk (Desai et al., 2003; Ouedraogo et al., 2008), or a protective effect of iron supplementation (Zlotkin et al., 2013). However, many of the studies that followed the Pemba trial were smaller in scale and/or introduced measures to combat malaria, such as bednets or intermittent preventative chemoprophylaxis, which may have masked any increase in malaria susceptibility.

Two Cochrane reviews have since been published in attempts to reconcile the apparently disparate findings of the Pemba study and subsequent trials (Ojukwu et al., 2009; Okebe et al., 2011). Both reviews concluded that iron supplementation did not increase the risk of malaria infection in children when “regular malaria surveillance and treatment services” were provided. However, many areas of the world have inadequate malaria surveillance and treatment, coupled with high levels of iron deficiency and anemia. We would posit that two major questions remain unanswered by the current literature.

First, to what degree must malaria be controlled, or treatment and prevention practices put in place, before the benefits of giving iron outweigh the risks? This question remains practically difficult to answer due to the ethical difficulties inherent in providing iron supplementation without introducing malaria-reduction measures, but further guidance is required for clinicians, nutritionists, and policymakers.

A second and related question is: should iron supplementation be restricted to those children who are anemic and/or iron deprived? The updated Cochrane review found that the benefits of iron supplementation were greatest among children who had the lowest hemoglobin levels at baseline (Okebe et al., 2011). Not only might these children reap the greatest benefits from supplementation, they may also be less likely to exhibit increased malaria incidence or severity as a consequence of increased iron stores. In a substudy of the Pemba trial, in which children were more closely monitored and given malaria-reduction measures such as insecticide-treated bednets (ITNs), the authors did not find that iron supplementation associated with increased malaria incidence or mortality (Sazawal et al., 2006). Moreover, children in this substudy who were iron deficient and anemic at baseline showed a significant *reduction* in adverse events, including malaria episodes, when supplemented with iron and folic acid (Sazawal et al., 2006). This finding was apparently echoed by a subsequent study that examined the effect of food fortification (micronutrient powders with or without iron) on malaria risk in Ghanaian children (Zlotkin et al., 2013), which found that children who were both iron deficient and anemic at baseline showed reduced incidence of malaria following iron supplementation. However, as was mentioned in an editorial on this study (Prentice et al., 2013) in the Ghanian trial, the iron-containing micronutrient powders were not actually effective at reducing anemia, making these results challenging to interpret. Moreover, iron supplementation in this trial was found to be associated with increased hospital admissions during the intervention period.

Targeting iron supplementation toward iron-deficient or anemic children is complicated by both practical and fiscal obstacles. As previously stated, most indicators of iron status can be confounded by infection or inflammation, limiting their interpretation in the field, and the widespread use of such tests may be difficult to fund. A solution to the problem may be to find more efficient ways to avoid giving iron to individuals who are iron replete, infected, or at high risk of infection (Atkinson et al., 2014). For example, in many areas, malaria transmission and prevalence of iron deficiency is seasonal; iron supplementation given outside of the malaria season may be less likely to increase susceptibility to infection, although studies are required to assess whether sub-patent parasitemias during the dry season may be increased by iron supplementation and cause illness.

When considering supplementation guidelines in pregnant women, it should be considered that primigravidae are both more susceptible to malaria infection and less prone to iron deficiency. One study demonstrated that intravenous iron supplementation increased malaria frequency in primigravidae but not multigravidae (Oppenheimer et al., 1986). Studies focusing on the effects of randomized oral iron supplementation have produced differing results (Menendez et al., 1994; Nacher et al., 2003), but relatively few have been performed thus far. In a large cohort study, it was found that iron-replete primigravidae are significantly more likely to experience placental malaria, but this effect loses significance in multigravidae (Kabyemela et al., 2008; Friedman et al., 2009). Summarizing the available evidence, adjusted supplementation guidelines that recommend lower iron supplementation to primigravidae may be advantageous.

## HOW THE MALARIA PARASITE BENEFITS FROM IRON

Iron is a limiting factor for the growth of many bacterial or protozoan pathogens. Iron-chelating agents such as desferrioxamine have been shown to restrict malaria growth *in vitro* (Raventos-Suarez et al., 1982), in murine models of malaria infection (Fritsch et al., 1985; Ferrer et al., 2012), and in malaria-infected monkeys (Pollack et al., 1987) (reviewed in Mabeza et al., 1999). Consequently, iron-chelating agents have been considered as an adjunct or primary antimalarial therapy.

In humans, preliminary work on the use of the iron chelators desferrioxamine, or the orally administered deferiprone, as an adjunct to standard antimalarial therapy seemed promising (Traore et al., 1991). However, after further studies showed no clear benefit from administration of chelators, and one seemed to hint at a slight increase in mortality in the trial group treated with desferrioxamine (Thuma et al., 1998), a Cochrane review recommended that trials testing iron chelators for malaria treatment be discontinued (Smith and Meremikwu, 2003).

The development of antimalarial drugs that target the parasite’s access to iron might meet with more success if we had a better understanding of how *Plasmodium* parasites acquire iron during their various life stages. Blood-stage parasites have been theorized to acquire iron from serum transferrin (Rodriguez and Jungery, 1986), from iron produced during the breakdown of hemoglobin, or from a free pool of intracellular iron (Hershko and Peto, 1988), but this important issue remains unresolved. Very little work has been done that investigates the acquisition of iron by the obligate liver or mosquito life stages of malaria infection; these continue to be fruitful areas for future research. For example, identification of *Plasmodium*-encoded iron transporters would both increase our understanding of the mechanism by which different life stages obtain their iron, and provide new drug targets aimed at inhibiting parasite growth.

Iron repletion may also have effects on parasite growth through different mechanisms than direct utilization by the parasite. A full synopsis of the literature is beyond the scope of this review, but iron has many effects on the immune system (McDermid and Prentice, 2006). In malaria specifically, it has been suggested that parasitized red blood cells from iron-deficient hosts may be more efficiently phagocytized, based on evidence from a murine model (Matsuzaki-Moriya et al., 2011). Finally, some *Plasmodium* species, notably *P. vivax*, live preferentially in young reticulocytes. Reticulocyte production can be restricted in highly anemic hosts, and this has been proposed as a mechanistic explanation for a study that showed an inverse association between severe anemia and *P. vivax* infection (Manning et al., 2012).

## HUMAN IRON CONTROL: THE HORMONE HEPCIDIN

Systemic mammalian iron metabolism is controlled at the level of iron absorbance from the diet and iron recycling through macrophages. Approximately 1 mg of iron is absorbed from the diet every day, roughly equivalent to the iron that is lost daily in poorly regulated activities such as sweating, any bleeding, and the sloughing off of enterocytes. At the same time, iron already in the body is constantly being recycled as macrophages phagocytose senescent or damaged red blood cells, digest the heme and extract the iron they contain, and export that iron back into the circulation.

The export of iron across the basolateral membrane of enterocytes and the recycling of iron through macrophages are dependent on the same transport protein: ferroportin, the sole currently identified mammalian iron export protein (Abboud and Haile, 2000; Donovan et al., 2000; McKie et al., 2000). The release of iron from enterocytes into the bloodstream is the final step of absorption of iron from the diet. Hepcidin, discovered a decade ago by three groups working independently (Krause et al., 2000; Pigeon et al., 2001; Park et al., 2001), is a 25-amino acid protein that binds to ferroportin and causes it to be internalized and degraded (Nemeth et al., 2004). The effect of ferroportin inhibition by hepcidin is therefore to block uptake of dietary iron from the intestine, and to increase the accumulation of iron in macrophages. The result is a decrease in serum iron levels, which routes iron away from pathogens that could potentially exploit circulating iron, but may also render the host anemic by restricting iron availability to the erythron.

Evidence from both animal models and from human genetic lesions suggests that the hepcidin–ferroportin interaction is functionally non-redundant. Mice underexpressing hepcidin are severely iron-overloaded (Nicolas et al., 2001), mimicking the human genetic disorder hereditary hemochromatosis, which (in rare cases) is caused by genetic lesions in the hepcidin gene itself (Roetto et al., 2003), in genes that encode hepcidin-regulatory factors (Bridle et al., 2003), or by mutations in ferroportin that render it resistant to hepcidin control (Drakesmith et al., 2005). Conversely, mice that overexpress hepcidin are fatally anemic (Nicolas et al., 2002a), while patients with mutations that lead to chronic hepcidin overexpression suffer from iron-refractory iron-deficiency anemia (Finberg et al., 2008).

Hepcidin increases in response to high iron conditions (Pigeon et al., 2001), but it is also upregulated in response to infectious and inflammatory stimuli (Nicolas et al., 2002b). Hepcidin is controlled homeostatically primarily via the bone morphogenetic protein (BMP) pathway. BMPs signal through the phosphorylation of SMAD transcription factors, which bind to a well-studied site on the hepcidin promoter and increase hepcidin transcription (Wang et al., 2005). Inflammation and infection also induce interleukin (IL)-6 or IL-22, which can upregulate hepcidin through the phosphorylation of STAT3 (Nemeth et al., 2003; Armitage et al., 2011). BMP/SMAD signaling has also recently been linked to hepcidin upregulation in infectious or inflammatory conditions by the molecule activin B, another member of the transforming growth factor β (TGFβ) superfamily that is upregulated by inflammatory stimuli but acts to upregulate hepcidin through SMAD signaling (Besson-Fournier et al., 2012). Hepcidin is suppressed by anemia, hypoxia, or erythropoietic drive, but, at the time of writing, the pathways underlying hepcidin suppression are less well articulated than are those involved in its upregulation.

## UPREGULATION OF HEPCIDIN IN MALARIA: CAUSES AND CONSEQUENCES

Hepcidin is upregulated in infections by bacterial, fungal, and viral pathogens (Armitage et al., 2011). Multiple studies have found that hepcidin is upregulated in malaria infection in symptomatic and asymptomatic natural human infections (Howard et al., 2007; de Mast et al., 2009a, 2010), in experimentally controlled human infections (de Mast et al., 2009b), and in murine models of malaria infection (Portugal et al., 2011; Wang et al., 2011). Resolution of infection leads to normalization of hepcidin levels (de Mast et al., 2009a).

Hepcidin’s upregulation in malaria has several important consequences. First, the upregulation of hepcidin leads to iron accumulation in macrophages and a decrease in serum iron, possibly contributing to the dyserythropoiesis and anemia that can accompany malaria infections. Additionally, hepcidin upregulation directly blocks dietary iron absorption: children with post-malarial anemia have high hepcidin levels and poorly incorporate orally administered iron into their red blood cells (Prentice et al., 2012).

The mechanisms whereby hepcidin is upregulated in malaria infection have yet to be fully characterized. IL-6 has been shown to be correlated with hepcidin in some studies (Casals-Pascual et al., 2012; Burte et al., 2013; Jonker et al., 2013), but in another study, urinary IL-6 and hepcidin were not significantly associated after a multiple, stepwise linear regression in infected humans (de Mast et al., 2009a). In one *ex vivo* study, human peripheral blood mononuclear cells co-incubated with *Plasmodium-*infected red blood cells showed a significant upregulation of hepcidin mRNA without concomitant IL-6 message increase (Armitage et al., 2009), but the relative contribution of peripheral blood mononuclear cells to systemic hepcidin levels is unknown. Finally, a study looking at the mechanisms by which blood-stage malaria infection can prevent the establishment of a liver-stage infection, which is thought to be modulated by hepcidin, found that liver-stage inhibition was preserved in mice treated with anti-IL-6 antibodies (Portugal et al., 2011). In brief, the role of IL-6 in hepcidin regulation in malaria remains controversial, and it is, as yet, unclear which other pathways may contribute to hepcidin upregulation in malaria infection.

Despite the paucity of knowledge on precisely how hepcidin is upregulated in malaria, the fact that both infection and iron repletion lead to increased hepcidin, and that increased hepcidin prevents oral iron absorption, suggests its potential utility as a point of care test to guide iron supplementation in areas of high infectious burden. Low hepcidin would indicate both the absence of infection and the probability of efficient iron absorption, while high hepcidin would indicate either adequate iron stores or ongoing infection. In the latter situation, iron supplementation would likely be unnecessary due to adequate iron stores, poorly absorbed as a direct result of high hepcidin, and/or potentially harmful if hepcidin is elevated due to an ongoing infection.

## UPREGULATION OF HEPCIDIN IN MALARIA: FOCUSING ON CO-INFECTION

Hepcidin has been recently shown to play a crucial role in determining the multiplicity of malaria infections within a single host. The obligate liver stage of the malaria parasite requires iron: hepcidin peptide injection or hepcidin overexpression by transgene or viral vector can reduce parasite survival at the crucial hepatic bottleneck (Portugal et al., 2011). The hepcidin upregulation initiated by one blood-stage infection thereby blocks the establishment of a second infection (Portugal et al., 2011).

**FIGURE 1:**
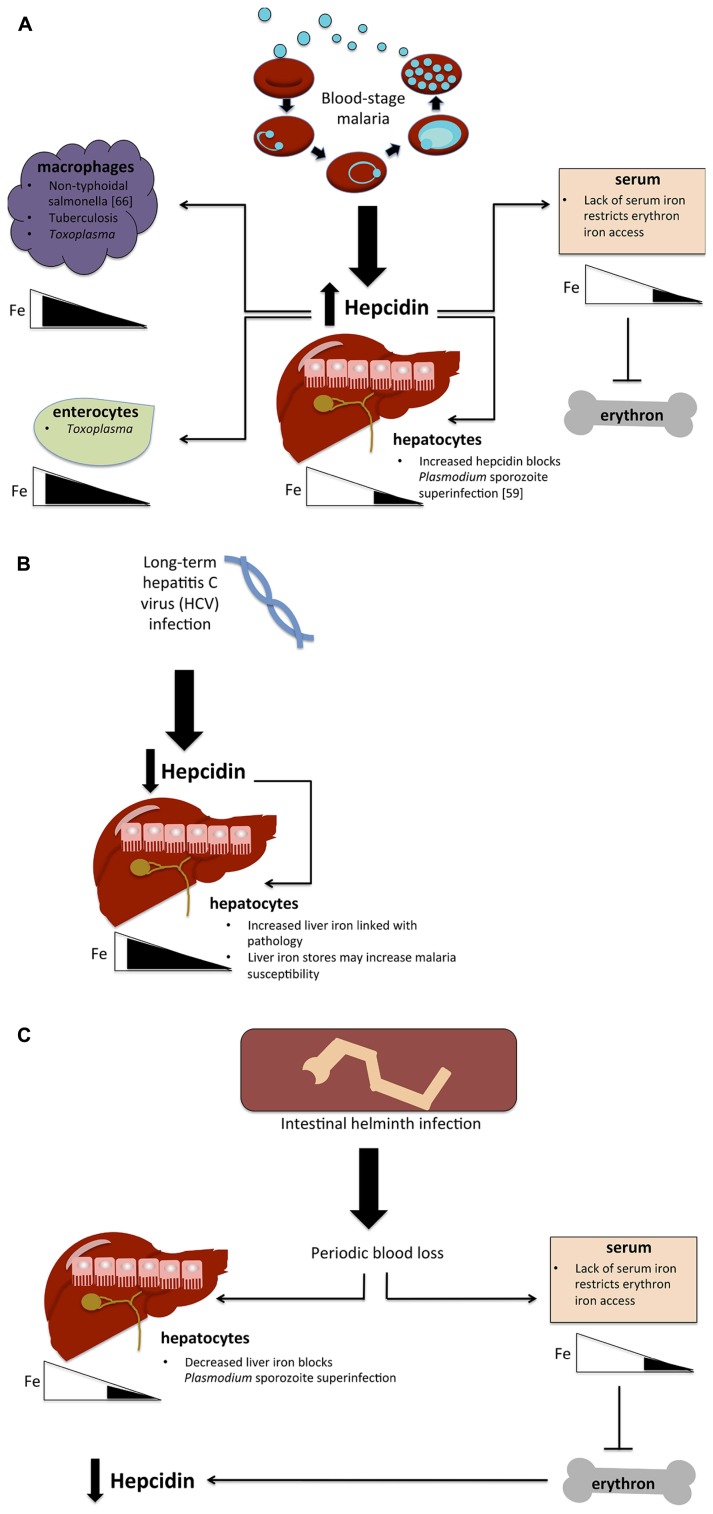
**Hypotheses for the effect of co-infections on iron metabolism. (A)** Possible implications of blood-stage malaria infection on host susceptibility to other infections. Blood-stage parasitemia causes hepcidin upregulation, which in turn routes iron away from hepatocytes and lowers serum iron levels, blocking the erythron’s access to iron and causing anemia. Lowered hepatocyte iron levels prevent the establishment of a second malaria infection (superinfection) by blocking liver-stage growth. Hepcidin also causes iron levels to increase in macrophages and potentially in enterocytes as well, thus possibly giving an advantage to pathogens that exploit the macrophage niche (non-typhoidal salmonella, tuberculosis, *Toxoplasma*) and those that require iron in enterocytes (*Toxoplasma*). **(B)** Hypothesized effect of HCV infection on malaria susceptibility. Long-term HCV infection causes hepcidin suppression, thus increasing liver iron stores and plausibly increasing malaria susceptibility. **(C)** Potential impact of intestinal helminth infection. Helminths cause periodic blood loss, which restricts iron to the liver, potentially blocking malaria liver-stage infection as in **(A)**. Decreases in serum restrict iron availability for erythropoiesis, causing anemia, which in turn causes the downregulation of hepcidin.

The physiological redistribution of iron as a consequence of hepcidin upregulation may also have a significant effect on host susceptibility to other bacterial, viral, or protozoa parasites (see **Figure 1**). In a blood-stage malaria infection, raised hepcidin is expected to contribute to increased macrophage iron levels, as does increased erythrophagocytosis. This increase in bioavailable macrophage iron may benefit pathogens that exploit the macrophage niche (van Santen et al., 2013). In particular, hepcidin upregulation may help to explain the association between malaria infections and susceptibility to non-typhoid salmonella (NTS). The epidemiological link between malaria and NTS is well established (Mabey et al., 1987). Iron has been implicated in the contribution of malaria to NTS susceptibility through increases in both free heme and heme-oxygenase expression (Cunnington et al., 2012). By routing iron to accumulate in macrophages, the hepcidin response to malaria may also render the host more vulnerable to NTS directly (van Santen et al., 2013). Similarly, tuberculosis could conceivably benefit from the increased iron availability in its macrophage niche, but the specific role of iron and hepcidin in this important co-infection has not been examined at the time of writing.

An important question is whether malaria-induced alteration of iron metabolism may modulate susceptibility to viral infections and vice versa. Malaria infections are associated with higher HIV viral load (Kublin et al., 2005). In turn, HIV-infected individuals are at greater risk of malarial infection (Patnaik et al., 2005). HIV affects iron metabolism in a complex manner: HIV is commonly associated with anemia, and in one study, hepcidin levels were shown to be elevated in HIV infection and inversely correlated with CD4 counts (Wisaksana et al., 2013). Conversely, increased iron stores in HIV-infected individuals are associated with mortality (McDermid et al., 2007, 2009) and risk of tuberculosis infection (McDermid et al., 2013). In an HIV-infected host, how do perturbations in iron and hepcidin modulate host susceptibility to malaria? Interestingly, HIV antiviral drugs have been found to demonstrate anti-*Plasmodium* activity at asexual (Skinner-Adams et al., 2004), liver (Hobbs et al., 2009), and gametocyte (Hobbs et al., 2013) life stages. However the effect of combination therapy on *Plasmodium* in HIV-1-positive patients, and the interactions of anti-retrovirals with anti-malarials and their overall effect on iron metabolism are difficult to predict.

Chronic hepatitis C virus (HCV) infection leads to hepcidin suppression and concomitant hepatic iron overload (Girelli et al., 2009). Might this increased iron lead to increased malaria susceptibility? Exposure of mice to HCV RNA induces a type 1 interferon response that has the effect of reducing parasite liver infection 48 h later (Liehl et al., 2014), but a long-term productive HCV infection and consequent iron overload may affect malaria risk very differently. More studies are needed to establish how chronic viral infections alter iron metabolism and malaria susceptibility.

Another unresolved issue is the effects of malaria on toxoplasmosis susceptibility. *Toxoplasma gondii* is carried asymptomatically by a large proportion of the human population, and it can cause life-threatening infections in pregnant women and the immunosuppressed. *Toxoplasma* infects hosts via enterocytes and can live in host macrophages (Hunter and Sibley, 2012). One study has shown that interferon gamma causes the death of *Toxoplasma* in enterocytes in a manner dependent on the depletion of intracellular iron stores (Dimier and Bout, 1998). Upregulation of hepcidin by a malaria infection would likely route iron toward enterocytes and macrophages, thus plausibly increasing host susceptibility and/or tolerance of *Toxoplasma*. If *Toxoplasma* does require intracellular iron stores to withstand the host response, then hepcidin upregulation and subsequent routing of iron to macrophages and enterocytes might increase host susceptibility to *Toxoplasma* infection.

A major cause of iron deficiency and anemia in the developing world is intestinal helminth infection. Infection with helminths is strongly associated with anemia in children (Calis et al., 2008; Jonker et al., 2012a); and de-worming treatment at the population level decreases anemia levels (reviewed in Smith and Brooker, 2010). Complex data exist on the contributions of intestinal helminth infections to malaria susceptibility and severity (Salgame et al., 2013). Early studies claimed that high-population helminth infection was associated with a striking lack of malaria, which the authors suggested was due to the nutritional perturbations associated with the worms (Murray et al., 1977). This finding was corroborated by more recent evidence suggesting that helminth infection in young children may be associated with fewer and later malaria episodes (Lyke et al., 2005), or protection from some of the potentially severe manifestations of malaria infections (Nacher et al., 2001, 2002). These effects remain controversial; further work has shown associations between helminths and higher parasitemia (Degarege et al., 2012) or severe malaria (Le Hesran et al., 2004). A single study found that infection with filarial worms ameliorated the drop in hemoglobin and reduced inflammatory cytokine production during malaria infection (Dolo et al., 2012). The impact of helminth infections on hepcidin is not well established, but it is plausible that helminth infection may, through periodic intestinal bleeding, cause constitutionally low hepcidin, as the body attempts to recoup the lost iron. At the same time, by causing iron deficiency, helminth infection may decrease host susceptibility to malaria or accentuate the effects of a hepcidin increase in infection.

Most laboratory studies on iron/infection interactions to date have focused on the effect of a single pathogen. Throughout human history, however, as well as in developing nations where infectious disease is still highly prevalent, susceptible individuals are frequently prey to multiple pathogens simultaneously. Understanding how pathogens affect iron metabolism and thereby modulate host susceptibility to other infections is likely to prove important both for its implications on treatment and on public health recommendations.

## SUPPRESSION OF HEPCIDIN IN SEVERE MALARIA SYNDROMES

Although the majority of studies on hepcidin and malaria have demonstrated an upregulation of hepcidin in malaria infection, three recent studies have shown that in certain circumstances, hepcidin suppression may also occur. One study found that among all children presenting with malaria, those with severe anemia had the lowest hepcidin levels (Casals-Pascual et al., 2012). A further study (Burte et al., 2013) demonstrated that children with uncomplicated malaria had higher hepcidin levels than those who could be classified as either presenting with severe anemia (in all studies cited, severe anemia was defined as Hb ≤5 g/L) or cerebral malaria. Finally, a group of children with severe malarial anemia exhibited very low hepcidin serum levels (over 50% were undetectable by the study’s method; Jonker et al., 2013).

Taken together, these studies clearly indicate that in severe malarial anemia, the signaling pathway that suppresses hepcidin can override the activation pathways associated with parasitemia. The mechanisms of hepcidin suppression by erythropoietic drive, hypoxia, or iron deficiency have not yet been well defined, but some groups have posited the existence of a bone marrow-secreted factor that suppresses hepcidin during erythropoiesis (Ginzburg and Rivella, 2011). In the two human studies that compared erythropoietin levels with serum hepcidin in malaria infection, erythropoietin and hepcidin were negatively associated (Casals-Pascual et al., 2012; Jonker et al., 2013). One animal study has also demonstrated a significant negative correlation between serum erythropoietin and hepcidin liver message in mice infected with *P. berghei* (Wang et al., 2011).

Further studies utilizing animal models of severe malarial anemia will likely be required to explore this aspect of iron control in malaria infection. In addition, the role of iron and hepcidin in cerebral malaria requires investigation.

## MALARIA PREVENTION EFFORTS REDUCE ANEMIA PREVALENCE

A recently published meta-analysis of global anemia prevalence estimated that in 2010, the prevalence of anemia was 32.9%, accounting for 8.8% of total years of life lived with disability worldwide (Kassebaum et al., 2014). In malaria-endemic countries, malaria is a major contributor to anemia at the population level: the authors estimated that in sub-Saharan African, 24.7% of anemia is attributable to malaria (Kassebaum et al., 2014). Therefore, interventions that reduce the prevalence of malaria could be expected to result in a reduction in the severity and prevalence of anemia.

The global community is currently using multiple complementary tools to reduce malaria at the population level. Intermittent preventative treatment (IPT) refers to the practice of administering antimalarial drugs presumptively during the especially vulnerable periods of pregnancy or early childhood. Insecticide-treated bed nets (ITNs) reduce nocturnal bites from *Anopheles* mosquitoes (Hill et al., 2006). Both interventions serve to protect the recipient and to reduce transmission at a population level (Greenwood, 2004).

A recent Cochrane review examining the effects of IPT for children under 5 concluded that of five West African trials examined, there was likely a significant reduction overall in moderate anemia incidence in areas with seasonal *P. falciparum* malaria transmission (Meremikwu et al., 2012). Of the two trials, which considered severe anemia as a separate outcome, both also found a significant reduction as a result of IPT (Dicko et al., 2011; Konate et al., 2011). This is consistent with the findings of a previous Cochrane review, which had also concluded that IPT in pregnant women was similarly associated with decreased rates of both severe anemia and all anemia (Garner and Gulmezoglu, 2006).

Fewer studies have examined the effects of the introduction of ITNs only on anemia prevalence, but earlier introduction of ITNs is associated with a statistically significant reduction in anemia prevalence in children at 6–12 months of age (Muller et al., 2006). In another study, hemoglobin levels were found to be significantly associated with the use of ITNs in young children (Holtz et al., 2002).

Why does reducing malaria infection have such a profound effect on population anemia levels? The relationship between malaria control and anemia risk may be partially dependent on hepcidin and its effects on iron absorption and utilization (as well as by reducing the well-known inhibitory effects of malaria on erythropoiesis). The hepcidin increases associated with malaria infection prevent efficient iron uptake. Iron supplementation has been shown to be less effective in areas with high malaria transmission (Gera et al., 2007).

Two studies have delineated the effects of malaria infection and hepcidin increase on the incorporation of orally administered iron into erythrocytes (Doherty et al., 2008; Cercamondi et al., 2010). In a study of young Beninese women, asymptomatic parasitemia was associated with poor incorporation of orally administered iron into RBCs, an effect that did not extend to parentally administered iron. Treatment and resolution of parasitemia resulted in a decrease in serum hepcidin and improved absorption of oral iron supplements (Cercamondi et al., 2010).

Similarly, young children with post-malarial anemia were shown to poorly absorb orally administered iron supplements (Doherty et al., 2008). Hepcidin levels in sera of these children were measured subsequently; these hepcidin levels were shown to be the best predictors of iron absorption (Prentice et al., 2012). Furthermore, despite poor oral absorption of iron, these children showed a more rapid hematological recovery than anemic and aparasitemic children, suggesting that hepcidin upregulation in malaria may contribute to relocalization of iron to macrophages, rather than true iron deficiency.

Populations with a high prevalence of malaria infections may therefore suffer both from anemia as a direct consequence of malaria infections, but also from poor utilization of oral iron as a result of chronically upregulated hepcidin.

## DISCUSSION AND RECOMMENDATIONS

Anemia continues to be one of the most common causes of disability worldwide; while *Plasmodium* is one of the most prevalent human pathogens. The relationship between the two is complex: low iron status may protect against malaria infection, but malaria infection in turn is linked with anemia at both the individual and population levels. New clues to this relationship have been obtained through our improved understanding of iron metabolism.

Specifically, the relatively recent identification of the human iron hormone hepcidin has allowed us to begin to explore these relationships in more depth. Hepcidin is upregulated in malaria infection, likely contributing to anemia through the relocalization of iron to macrophages and the prevention of iron uptake from the diet. However, in certain severe malaria syndromes, hepcidin may be suppressed. The mechanisms behind both hepcidin’s increase and its suppression in malaria are currently unclear.

At the moment, action on three fronts is required. First, we must implement our improved understanding of the iron–malaria relationship toward optimizing malaria prevention and anemia treatment. Research findings support more nuanced iron supplementation regimens, rather than the blanket approach of supplementing, or not supplementing, whole populations. Supplementation could be scheduled around the malaria season (Atkinson et al., 2014), routed to the most iron-deprived children or pregnant women, or targeted based on hepcidin levels as a proposed biomarker (Pasricha et al., 2014). At the very least, the findings of the last decade of research since the Pemba trial argues very strongly that iron supplementation should be carried out in concert with malaria control efforts; and that improved malaria control may itself be effective as a means of decreasing anemia at the population level.

Second, clinically focused research is required to develop better and rationally informed therapeutics for those who become infected. For example, understanding the mechanisms underlying hepcidin upregulation in malaria could be the first step toward the development of drugs that, given concurrently with antimalarial treatment, repress hepcidin and speed recovery from anemia. Carefully designed *in vivo* and *in vitro* studies could explore the role that hepcidin may play in mediating the outcomes of co-infections between malaria and other protozoan, bacterial, or viral pathogens, providing vital information for treating individuals and populations exposed to multiple pathogens simultaneously.

Finally, in the longer term, basic research should focus on an improved understanding of the host–pathogen tug-of-war over iron metabolism. How does malaria acquire iron at a molecular level in its many life stages? Do malaria pathogens show different phenotypes in iron-deprived hosts? Armed with new tools to explore pathogen biology and iron metabolism, we have a chance to answer questions of immediate clinical importance, and to advance the basic science behind our understanding of the host–parasite relationship.

## Conflict of Interest Statement

The authors declare that the research was conducted in the absence of any commercial or financial relationships that could be construed as a potential conflict of interest.

## Footnotes

1 http://www.who.int/malaria/publications/world_malaria_report_2013/en/
